# The Curse of Opiates: A Lay Opinion

**Published:** 1887-12

**Authors:** 


### THE CURSE OF OPIATES: A LAY OPINION.

"The sad case of the beautiful actress, Sara Jewett, a victim to the deadly and insidious 'Chloral habit,' points a moral which he who runs may read. The true story seems to be that some years ago, in London, Miss Jewett, broken down from overwork and loss of sleep, consulted a prominent English physician, who prescribed a compound of orange-flower water and Chloral.

"The prescription brought immediate relief, and the first step taken, the rest was easy, until Miss Jewett, like so many other talented men and women, alas! became a slave to the dangerous and deadly drug. It is to such people that the Chloral and Morphia habits are most dangerous. Overwrought nerves are quieted, overtaxed brains are soothed, and not infrequently in total ignorance of what they are doing, *relying blindly on the physician* who has given them such swift relief, they turn to it again and always, until existence becomes a burden without it, and the well-nigh incurable habit is firmly fixed.

"'There is nothing which I can give you to relieve you absolutely except Chloral,' once said an eminent physician to a patient in the agonies of neuralgia, brought on by nervous prostration, 'and my advice to you is, suffer anything rather than take it; it is the most insidious devil in the world.'

"The physician is too often to blame for the formation of the habit, since a warning like that just repeated would save many. * * * It were better for man or woman that 'a millstone were hanged about their necks and they were cast into the sea' than that they should fall under the power of this curse of the nineteenth century."—*The Phila. Daily News, Oct. 8th.*

("Relying blindly on the physician," says the *News.* Alas!

what is the result? Patients become victims to this "curse of the nineteenth century." Many a doctor will hereafter answer to his God for a father or a mother whom he has so carelessly enslaved, and whose helpless children cry aloud for vengeance. And yet Deans of *homœopathic* medical schools recommend the use of opiates!)

